# Phylogenetic analysis of rabies surveillance samples from north and northeast Brazil

**DOI:** 10.3389/fvets.2023.1257558

**Published:** 2023-09-29

**Authors:** Tânia Cristina Alves da Silveira da Cunha, Fábio Silva da Silva, Sandro Patroca da Silva, Ana Cecília Ribeiro Cruz, Francisco Amilton dos Santos Paiva, Livia Medeiros Neves Casseb, Ana de Nazaré Silva do Nascimento, Iza Alencar Sampaio de Oliveira, Marlon de Araújo Castelo Branco, Rodrigo Adolpho Brasil de Oliveira, Darlene de Brito Simith Durans, Thito Yan Bezerra da Paz, Taciana Fernandes Souza Barbosa Coelho

**Affiliations:** ^1^Department of Arbovirology and Hemorrhagic Fevers, Evandro Chagas Institute, Ananindeua, PA, Brazil; ^2^Technical Management of Environmental Health Surveillance, Health Surveillance State Agency, Porto Velho, RO, Brazil; ^3^Technical Area of Zoonoses and Venomous Animals, Tocantins State Health Secretariat, Palmas, TO, Brazil; ^4^Laboratory of Rabies, Zoonosis Control Management, Teresina, PI, Brazil; ^5^Central Laboratory of Roraima, Boa Vista, RR, Brazil

**Keywords:** rabies, surveillance, *Lyssavirus*, genotypes, bats, variants, northern Brazil

## Abstract

Viruses of the *Lyssavirus* genus are classified into several genotypes (GT1 to GT7), of which only GT1 (classic rabies virus—RABV) has a cosmopolitan distribution and circulates in Brazil. GT1 is subdivided into several antigenic variants (AgV) maintained in independent cycles with a narrow host range and distinct geographic distributions, namely, AgV1 and AgV2 found in dogs, AgV3 in the vampire bats *Desmodus rotundus*, and AgV4 and AgV6 in bats non-hematophagous *Tadarida brasiliensis* and *Lasiurus cinereus*, a common variant of marmoset (*Callithrix jacchus*), and crab-eating fox (*Cerdocyon thous*). In this study, we performed phylogenetic analysis to identify at the antigenic variant level; six RABV genomes derived from the Rabies Surveillance in the north and northeast regions of Brazil. The analysis resulted in the formation of 11 monophyletic clusters, each corresponding to a particular variant, with high bootstrap support values. The samples were positioned inside the AgV3, AgV6, and *Callithrix* variant clades. This is the first report of the AgV6 variant found in northern Brazil, which provides valuable information for rabies surveillance in the country. The possibility of viral spillover has been much debated, as it deals with the risk of shifting transmission from a primary to a secondary host. However, more genomic surveillance studies should be performed, with a greater number and diversity of samples to better understand the transmission dynamics of each variant to detect changes in its geographic distribution and spillover events.

## 1. Introduction

Bats (order Chiroptera) are the main reservoir hosts for most viruses of the *Lyssavirus* genus (1). Species assigned to the genus are associated with acute progressive encephalomyelitis. Among them, the best-described species belong to *Lyssavirus* rabies, which is a disease that affects mammals. Lyssaviruses have different geographic distributions in different parts of the world; however, only the *Rabies virus* (RABV) has a cosmopolitan distribution, except for Antarctica and some isolated islands (2, 3).

Together with carnivores (order Carnivora), bats maintain the circulation of RABV. Rabies is transmitted directly between susceptible individuals by bites, scratches, or infection of mucous membrane virus-containing saliva, without the participation of arthropod vectors (2, 3).

Currently, 17 species are assigned to the *Lyssavirus* genus, which is divided into phylogroups I and II, according to their antigenic and genetic characteristics. Three species (*L. lleida, L. ikoma, and L. caucasicus*) are not included in the phylogroups, although they are well defined within the genus (4). These species are distributed into seven genotypes (GT1 to GT7), and only GT1, which includes the classic RABV, is of epidemiological importance given its association with a greater number of human cases of encephalomyelitis compared with other genotypes (2, 4).

GT1 has several antigenic variants associated with different animal species and regions or countries of origin. Animal hosts play a fundamental role in the maintenance of each of the RABV variants, which exist in nature in independent cycles, such as those related to hematophagous, frugivores, insectivorous bats, and wild canids, among others (2).

Seven antigenic variants have already been isolated in Brazil as follows: AgV1 and AgV2 found in dogs; AgV3 isolated from the vampire bat *Desmodus rotundus*; and AgV4 and AgV6 isolated from non-hematophagous bats, *Tadarida brasiliensis* and Lasiurus cinereus, common marmoset variant (*Callithrix jacchus*), and crab-eating fox variant (*Cerdocyon thous*) (2–4).

It is well established that certain variants are associated with specific animal species. Therefore, the distribution of a viral population is conditioned by the regional variation in the distribution of the host species, as well as by its biological aspects. The interaction between hematophagous and non-hematophagous bats and other wild animals facilitates the sharing of antigenic variants given their adaptation to certain regionally distributed secondary wild hosts (4).

Another widely discussed factor of great epidemiological relevance is the real and imminent possibility of changing the dynamics of viral transmission from a primary host, such as non-hematophagous bats, to secondary hosts, such as dogs and cats. Therefore, the prolonged maintenance of virus circulation in a new population can lead to the expansion of its geographic distribution, which justifies the introduction of new variants (3, 4).

### 1.1. Author summary

Rabies is a neglected disease directly related to socioeconomic factors. Rabies remains a worldwide public health problem due to its fatal evolution and the high number of human cases leading to a large number of people submitted to post-exposure treatments annually. The real magnitude of this problem is still unknown since epidemiological surveillance systems provide insufficient information to accurately measure the current scenario of the disease. Here, we report the circulation of three RABV variants and their epidemiological importance in the north and northeast regions of Brazil, which harbor a large diversity of hosts, including bats.

## 2. Materials and methods

### 2.1. Sampling location

**Figure 1:**
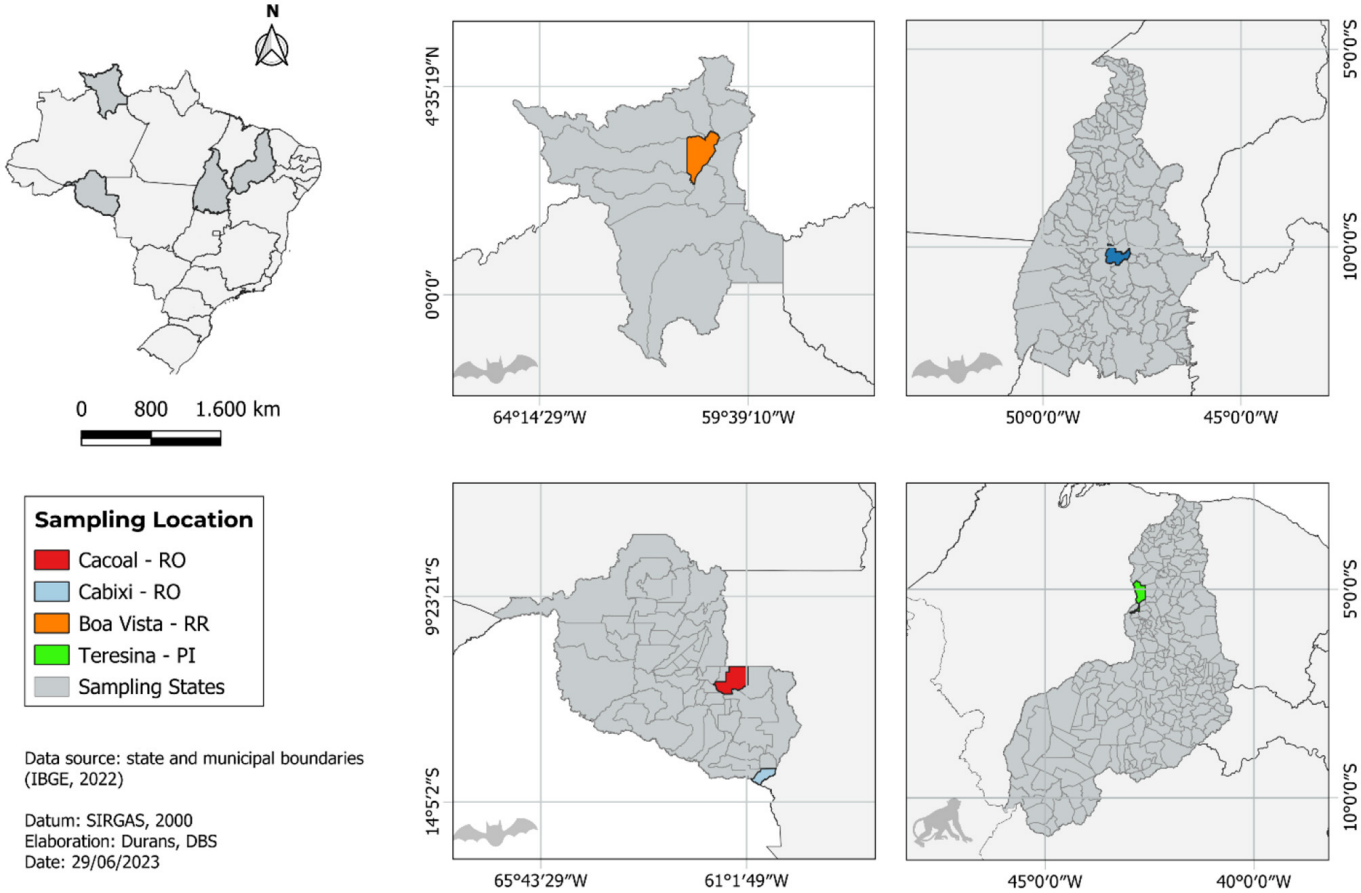
Map showing all locations of sample collection that have been analyzed in this study.

Six tissue samples collected from six animals captured in the north and northeast regions of Brazil were the source material for sequencing. Three of them came from Cabixi (n = 1, *Artibeus lituratus*) and Cacoal (*n* = 2, *Molossus molossus*) municipalities, Rondônia (RO) state. Two samples came from *A. literatus* captured in Palmas and Boa Vista, cities of Tocantins (TO) and Roraima (RR) states, respectively. From the northeast region, we obtained a sample from a non-human primate (NHP) of the genus *Callitrix sp*., from the city of Teresina, Piauí (PI) state (Figure 1). The samples came from routine surveillance to investigate possible cases of rabies in these areas, with no need for submission and approval by the Ethics Commission on Animal Use (CEUA).

### 2.2. RNA extraction, double-stranded cdna synthesis, and genomic library preparation

Approximately 10 mg of brain tissue was added to a 2 ml microtube together with 1 ml of TRIzol™ reagent and a 5-mm diameter steel bead and macerated in a TissueLyser II equipment for 2 min at a frequency of 30 Hz. Subsequently, 200 μl of chloroform was added, and the material was transferred to a tube containing a phase separator polymer, Phasemaker™ Tubes (Thermo Fisher Scientific), and kept at room temperature for 15 min. After centrifugation at 12,000 g at 4°C for 5 min, ~560 μl of the upper precipitation phase was recovered and transferred to a new tube, and 560 μl of pure ethanol was added. Total RNA from the samples was, then, purified using the PureLink RNA Mini Kit (Invitrogen Life Technologies), following the manufacturer's protocol starting with the column purification step described in the kit. After purification, the RNA was quantified using the Qubit RNA HS Assay Kit (Thermo Fischer Scientific) in the Qubit 4.0 equipment, according to the manufacturer's recommendations.

The synthesis of the first and second strands of cDNA was performed using the SuperScript TM VILO TM Master Mix Kit (Thermo Fisher Scientific, Waltham, MA, USA) and NEBNext^®^ Second Strand Synthesis Module (New England Biolabs, Ipswich, MA, USA), respectively. The cDNA purification reaction was performed with the PureLink^®^ PCR Purification Kit (Invitrogen Life Technologies). All steps, from cDNA synthesis to purification, followed the recommendations of the manufacturers. The synthesized cDNA was quantified with the Qubit Assay DNA HS Kit in the Qubit 4.0 equipment.

To prepare the genomic library, the Nextera XT DNA Kit (Illumina) guidelines were followed. Subsequently, the library was sequenced on the NextSeq 500 platform (Illumina) using the NextSeq 500/550 High Output Kit v2.5 (Illumina) (300 Cycles) and employing the paired-end methodology (2 x 150 bp), as recommended by the manufacturer.

### 2.3. Data analysis

Initially, the quality of the generated reads was evaluated using FastQC (5). Subsequently, DIAMOND software (6) was used to align the reads against the non-redundant (nr) protein database, taking into account the e-value (10–6) and amino acid identity. The output files generated by DIAMOND were converted into tabular output files, and these files were visualized in Krona v. 2.8 (7).

SortMeRNA v.2.1 was used to remove reads corresponding to ribosomal RNA (rRNA) (8). Next, Trim Galore v.0.4.5 (9) was used to remove short reads (<75 nt), adapters, and reads containing more than 10 undetermined bases and generate a FastQC file for the processed data.

The files generated in the data treatment step were assembled by the de novo method using SPAdes (10) (kmers: 21, 33, 55, and 77) and IDBA-UD (11) (kmers: 20, 40, 60, 80, and 100). The grouped contigs were aligned with DIAMOND under the same parameters described above.

### 2.4. Phylogenetic inference

A set of 44 nucleotide sequences corresponding to the nucleoprotein segment of RABV were analyzed. Of these, six were target sequences, five from the states in the north region (four from Rondônia state and one from Tocantins state) and one from the northeast region (Piauí state). Additionally, five of the target sequences are from bat samples and one from a non-human primate, from the years 2018, 2019, and 2021.

Using Geneious v.11.0 software (12), the nucleotide sequences were aligned using the MAFFT algorithm (13). Then, to evaluate the metrics of nucleotide distance between the sequences in the dataset, MEGA X software (14) was used, considering the *Maximum Likelihood Composition* substitution model. The final products were organized in a matrix of distances, which were later used for the analysis of intra/intergroup distances. The graphical representation was performed using R software (available at https://www.r-project.org/) together with the *ggplot2* libraries (available at https://ggplot2.tidyverse.org/), *reshape2* (https://cran.r-project.org/web/packages/reshape2/), and *pheatmap* (https://cran.r-project.org/web/packages/pheatmap/). Statistical means demonstrating the significance of the relationship between the assessed taxa were generated by applying Student's *t*-test (considering p < 0.05), with a 95% confidence interval and a sample error margin of 5%.

To evaluate the evolutionary selection pressure acting on the studied sequences, the ratios between the proportions of non-synonymous (*dN*) and synonymous (*dS*) substitutions (*dN/dS*) among the analyzed sequences were obtained using CodeML software (belonging to the PAML package) (15, 16). Additionally, to verify the existence of sites under diversifying selection pressure (positive) throughout the evaluated region, the online tool Datamonkey (17) was used, employing its analysis modalities BUSTED (Branch-site Unrestricted Statistical Test for Episodic Diversification) (18) and MEME (Mixed Effects Model of Evolution) (19).

Using the aligned sequence file, the phylogeny was reconstructed using the *Maximum Likelihood* method with IQ-TREE v.1.6.12 software (20). The software automatically defined the best replacement model based on the Akaike Information Criterion (AIC) (GTR+F+I+G4) and generated bootstrap values (BPP) based on 1,000 repetitions. An additional analysis was performed using the software to evaluate the phylogenetic signal of the sequence set based on the defined AIC model. Finally, the topology was visualized using Figtree software v.1.4.4 (21), with definition of the midpoint anchorage of the typology obtained by the midpoint method (22) and edited using Inkscape (available at: https://inkscape.org/pt-br/).

## 3. Results

**Figure 2:**
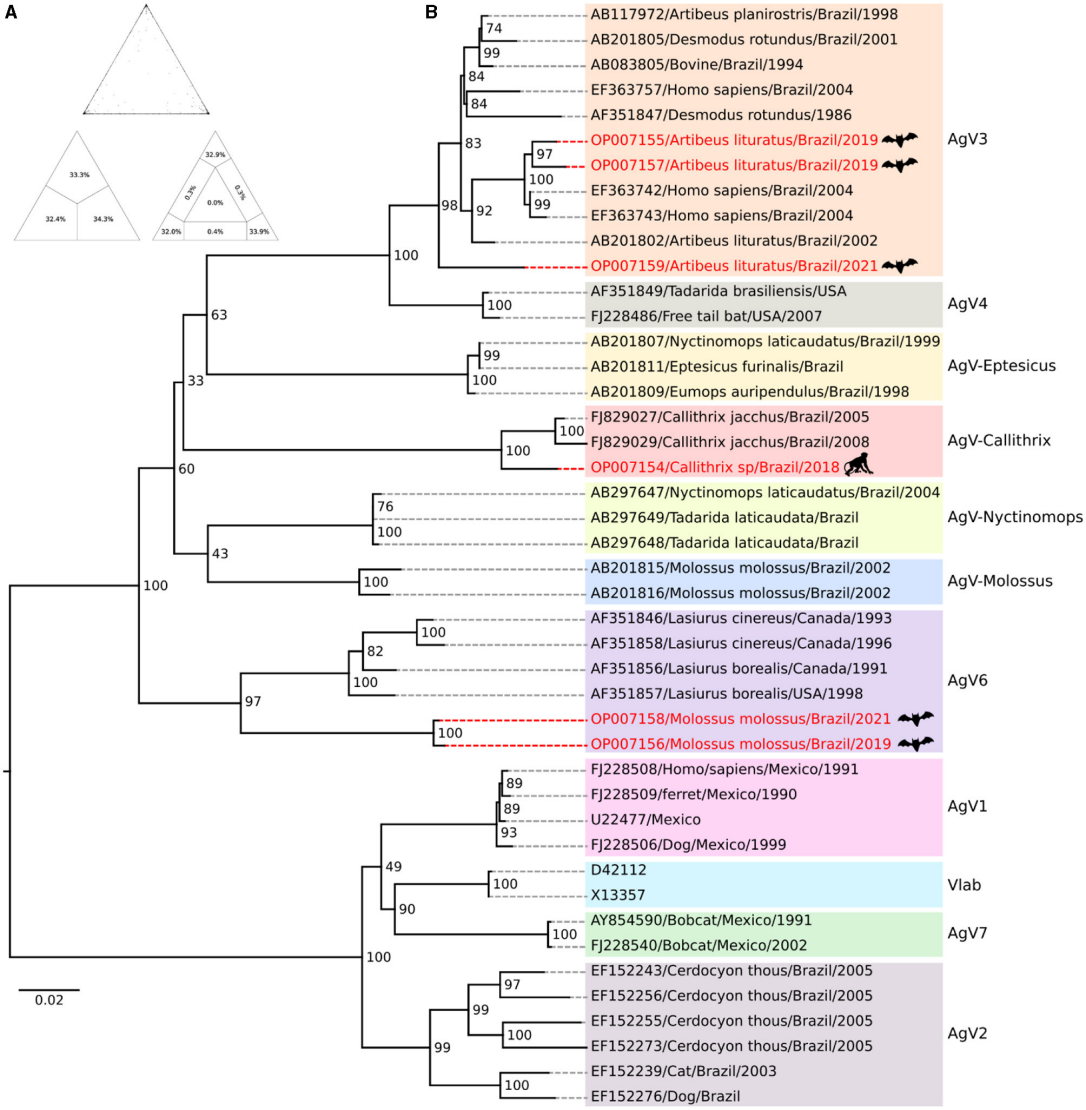
Phylogenetic inference of *Rabies virus*. **(A)** The value triangle shows a percentage representation of the analysis verifying the quality of the phylogenetic signal in the available sequence set which is shown in the value triangle. **(B)** The phylogeny is reconstructed using the *Maximum Likelihood* method. Bootstrap values are determined from 1,000 replicates and are indicated on each node. The highlighted red lines indicate the target taxa for the evolutionary positioning analysis.

Based on the sequence quartet evaluation methodology, the phylogenetic signal analysis resulted in a positive signal, with 98.8% of trees generated exhibiting high reliability and 1% of trees generated with medium reliability (Figure 2A). The presence of a negative phylogenetic signal, which is unsuitable for phylogeny reconstruction, is considered when the sum of unresolved and partially resolved regions exceeds 30%. Upon reconstructing the phylogeny, it was observed that 11 monophyletic groups were formed, demonstrating strong internal support. The target taxa were distributed among clusters that included sequences of VAg3 clade (taxa OP007155, OP007157, and OP007159) (BPP = 98), Variant *Callithrix* clade (taxon OP007154) (BPP = 100), and Variant 6 clade (taxa OP007158 and OP007156) (BPP = 98) (Figure 2B).

**Figure 3:**
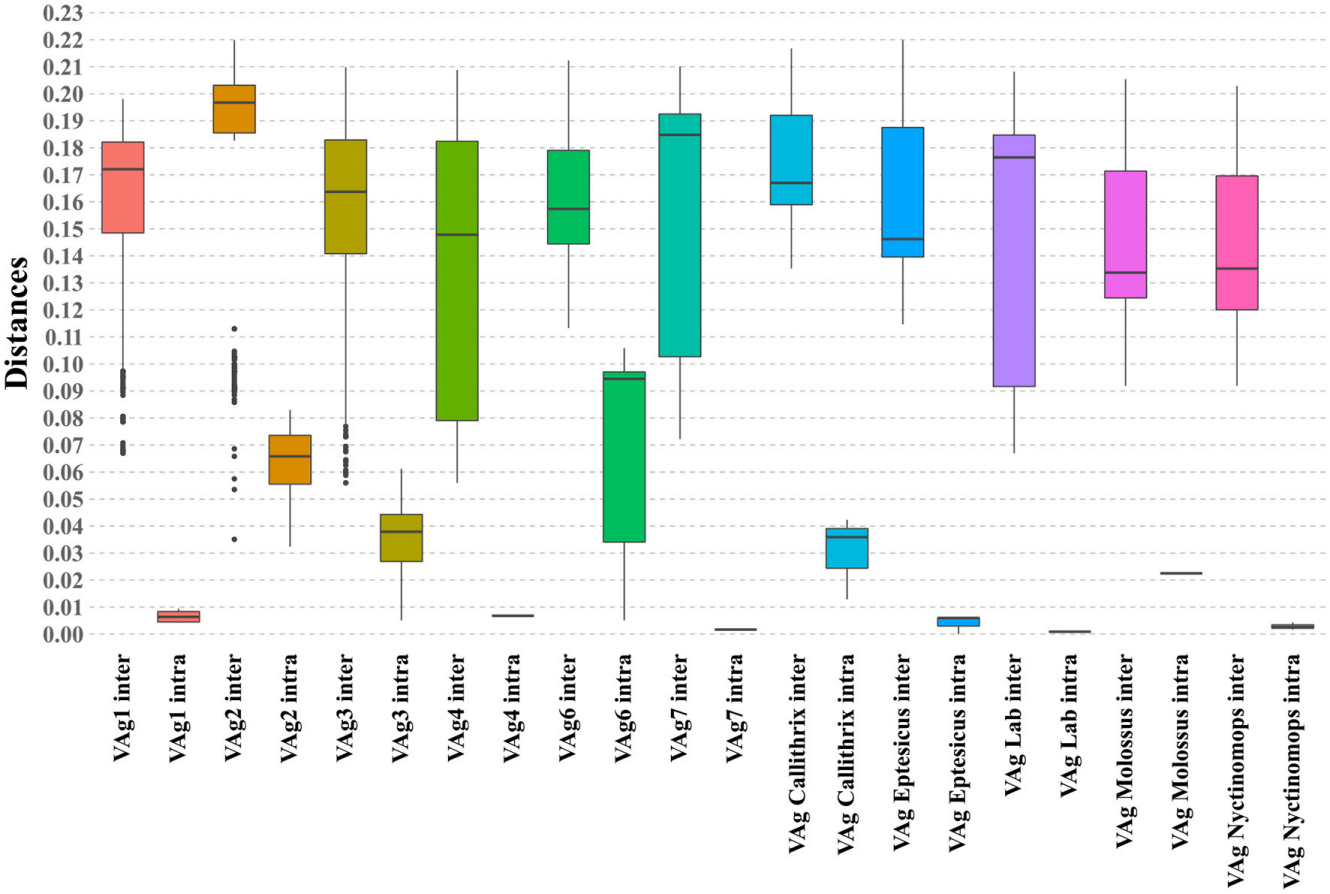
Boxplot graph illustrating the intragroup and intergroup distances of the phylogenetic grouping obtained, including the evaluated sequences. The analyzed clusters are indicated on the X-axis, and the Y-axis displays the observed percentages of nucleotide distance.

When evaluating the average nucleotide distances based on metrics using the *Maximum Likelihood Composition* model, an average distance of 0.14 was observed between the assessed taxa. Furthermore, in the evaluation of these distance metrics, significant differences (*p* < 0.05) were observed within the intra/intergroup comparative analysis when comparing the means obtained for the taxonomic grouping (Figure 3). These findings support the previous results regarding the formation of wellestablished taxonomic grouping among the evaluated variants as represented in the phylogenetic reconstruction analysis.

**Figure 4:**
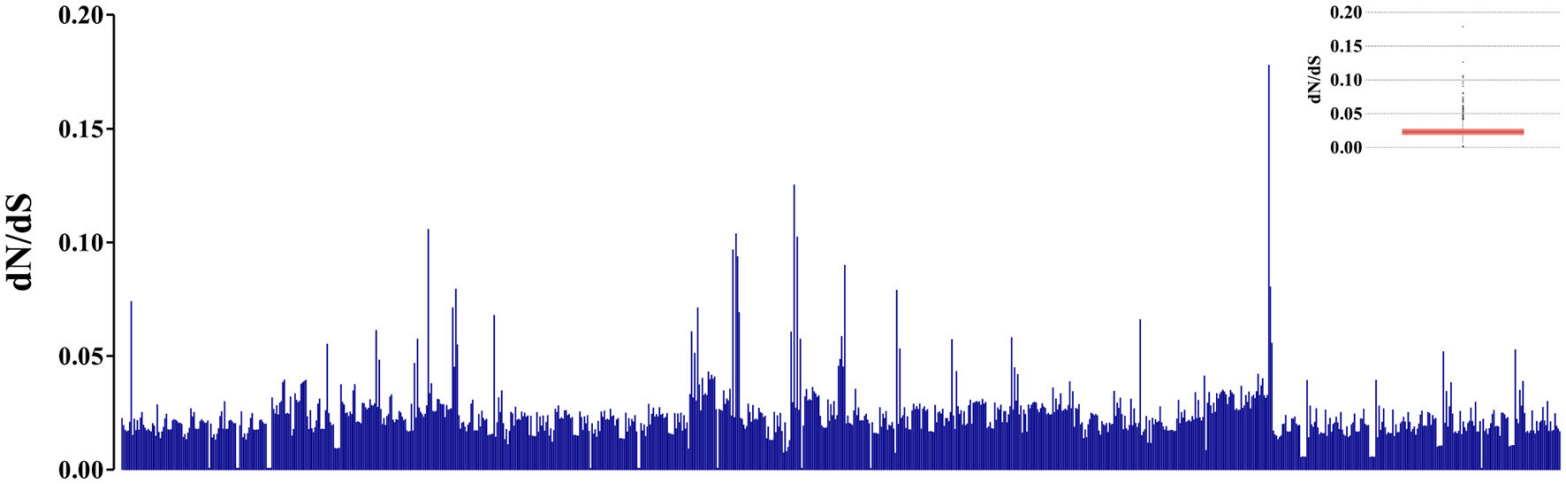
Graphic representation of 946 pairs formed by the analyzed sequences, illustrating the estimation of evolutionary pressure values using the *dN/dS* ratios. The Y-axis displays the indicative values of evolutionary pressure based on the *dN/dS* ratio. The internal boxplot graph indicates a prevalence of the *dN/dS* ratios between 0 and 0.05 for the evaluated pairs.

**Figure 5:**
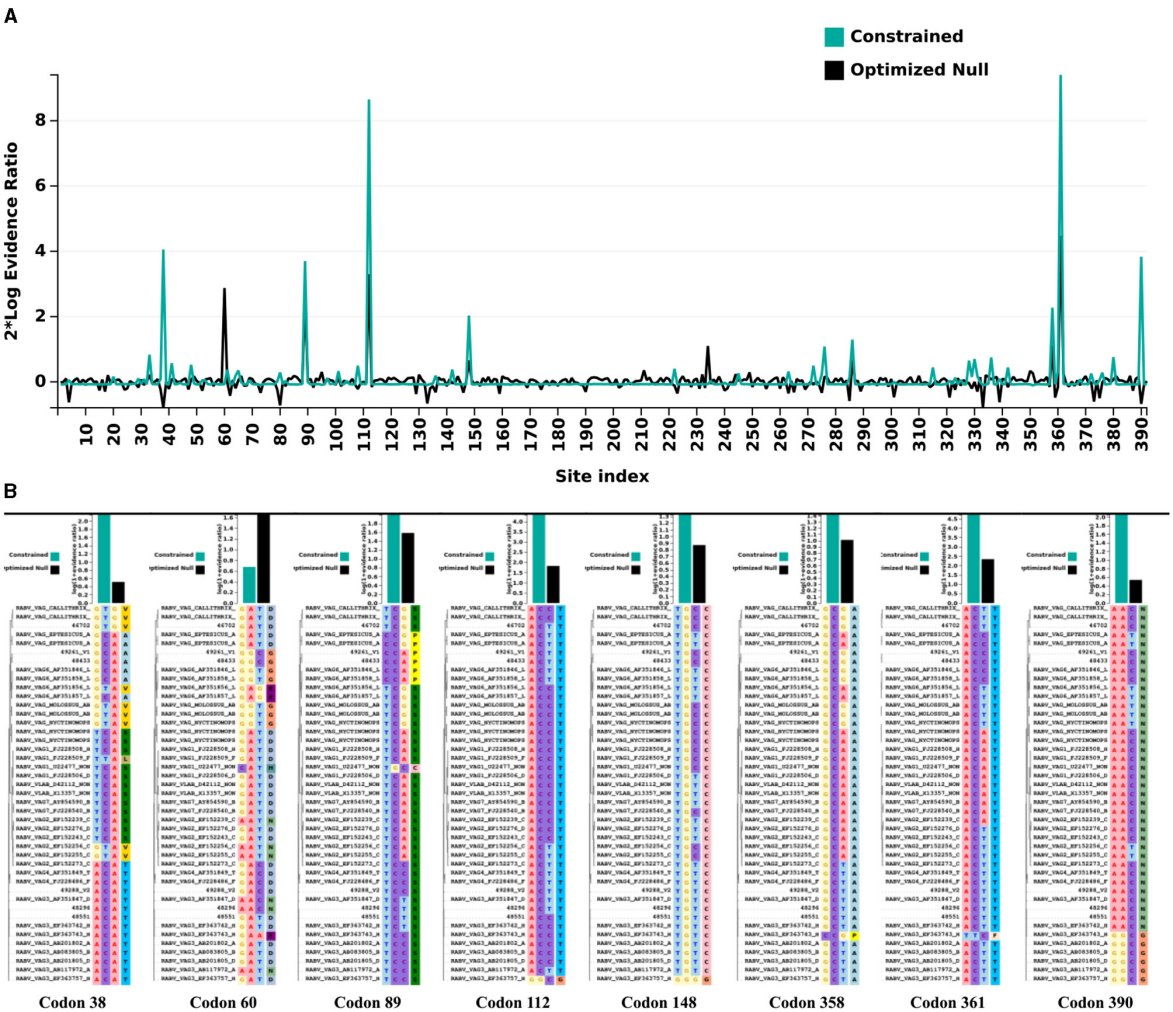
Evolutionary pressure analysis by *Datamonkey*. **(A)** Graphic representation illustrates the positions of sites along the evaluated gene region that experienced positive selection pressure effects. The numerical markings of the site positions are displayed on the X-axis, while the Y-axis represents the thresholds of evidence for positive selection pressure as defined by the tool used. **(B)** Sites (codons) along the evaluated region that exhibited diversifying (positive) selection effects, based on the defined parameters of the employed tool.

When analyzing the evolutionary pressure acting on the evaluated sequences, based on the non-synonymous to synonymous substitution ratios (*dN/dS*), it was observed that the studied gene region is undergoing global negative selection (purifying selection). This is indicated by the *dN/dS* ratios, which are consistently below 1, suggesting high levels of structural conservation. The range of the ratios is 0 ± 0.17 (Figure 4). Additionally, when examining the presence of sites under positive selection pressure in the set of evaluated sequences using the BUSTED tool, it is observed that the studied gene region, which evolves globally under negative selection pressure according to the previous analysis, still exhibits at least eight sites (Figures 5A, B) experiencing positive selection effects. The results obtained using the MEME tool are consistent with those from BUSTED, revealing an overall average *dN/dS* ratio of 0.0394 and detecting diversifying selection in at least one site within the evaluated region.

## 4. Discussion

In recent years, the reports of emerging *Lyssavirus* have become frequent, and these occurrences are facilitated by the great diversity of bats, mainly non-hematophagous ones, and their interaction between different bat species with other animals. This behavior favors spillover events facilitating the adaptation of viruses to new hosts, which contributes to the emergence of new viral strains with pathogenic potential for other animals and humans (2, 3).

RABV circulation was verified in the northeast region of Brazil in marmosets (*Callithrix jacchus*), corresponding to a unique antigenic group, unrelated to samples found in bats or terrestrial mammals. This reinforces the hypothesis that marmosets are a natural reservoir for the virus, since its reading profile is incompatible with any monoclonal antibody available for circulating variants in the USA, maintaining a unique pattern.

Regional genetic variations can also be observed in the northern region. A retrospective study characterized RABV samples from different mammal species, including humans, from different regions in the Pará state, isolated between the years 2000 and 2005, and suggested the circulation of at least three distinct genetic lineages of a variant associated with vampire bats, one of which is maintained in the Marajó region. This reinforces the hypothesis that the region, where the first major outbreak of human rabies transmitted by vampire bats occurred, constitutes a unique niche for the maintenance of the cycle related to these animals (23, 24).

The epidemiological profile of rabies has changed in recent years, and wild animals, including the various species of bats, are considered efficient transmitters of the disease to other animals and humans. Since 2015, human rabies occasioned by the canine variants, AgV1 and AgV2, has not been recorded, and the presence of non-hematophagous bats in urban areas, although protected by law due to their important ecological role, become a threat to the population, as the number of RABV positive species grows each year in Brazil (2, 25).

In the present study, five samples from non-hematophagous bats and one from an NHP from Rondônia, Roraima, and Tocantins in the north region and Piauí state, northeast region, were analyzed. Three sequences from non-hematophagous bats *Artibeus lituratus*, from Tocantins (OP007155), Rondônia (OP007157), and Roraima (OP007159) states, were grouped into the AgV3, clade AgV, although this variant is more frequently associated with *Desmodus rotundus* vampire bats.

The AgV3 variant is strongly established and dispersed in the north and northeast regions of the country (4). The presence of this variant in *A. lituratus* may be related to close interactions between hematophagous and non-hematophagous bats, which can, to a certain extent, live in proximity and share the same shelter. In such a manner, disputes over territory may facilitate the transmission of RABV variants between different species of bats (26).

In contrast to the sequences from Rondônia and Tocantins, the sequence from Roraima, although undoubtedly clustered with the AgV3 group, was the most divergent, positioned in the basal branch in the clade. This suggests that the genetic variability within the AgV3 lineage circulating in these regions is higher than previously known. However, further analyses with a larger number of sequences must be carried out to confirm this hypothesis.

The only sequence from an NHP sample (OP007154), from the Piauí state, was positioned in a wellestablished way in the *Callithrix sp*. variant clade, together with samples from other locations in the northeast region of the country. It is noteworthy that since the end of the 1980s, RABV has been isolated from NHPs, mainly from the white-tufted marmoset (*Callithrix jacchus*), in at least four states of that region (Rio Grande do Norte, Ceará, Piauí, and Pernambuco). In two of these states (Ceará and Piauí), human cases of rabies transmitted by marmosets were reported (27).

Marmosets became well adapted to the region and a more common source of infection for humans. Data from laboratory-confirmed cases in 20 years suggest the occurrence of regional transmission and a gradual increase in the geographical distribution, supporting the emergence of marmosets as a new reservoir for RABV. In addition, tourism, wildlife traffic, and the culture of keeping these animals as pets, especially in coastal regions, are the main risk factors for the increase in human cases of rabies (27).

As a result of multiple efforts, Brazil achieved satisfactory levels of control for urban rabies maintained by dogs and cats. However, rabies in wild animals is still a major challenge for public and private services focused on human and animal health. The scenario is patent in the north and northeast regions of Brazil, where several cases of rabies have been recorded in wild animal species (27, 28).

A great diversity of variants is found in non-hematophagous bats, which points to the existence of multiple independent transmission cycles, involving different species of bats. Isolates from several genera/species of bat, including the *Molossidae* family, have already been genetically characterized in studies from different regions of the country, which provided an important basis for the analyses of this study (2, 3, 23, 24, 29–32).

Surprisingly, the RABV sequences, OP007156 and OP007158, from the insectivorous bats, *Molossus molossus*, collected in 2019 and 2021, in the Cacoal municipality, Rondônia state (Supplementary Figure 1), were grouped into the AV6 clade, with sequences from *Lasiurus cinereus*. Several studies demonstrate a restricted distribution of AgV6 to the south and southeast regions of Brazil (31). This is the first description of the variant in the north region and detected in a species not characteristic of its lineage.

Bats from the *Molossus* genus are among those most frequently found infected with RABV, probably due to the ecological and behavioral characteristics of the genus, increasing their susceptibility to become infected by specific variants from other bats, as observed in other studies (31, 33, 34).

Similar events tend to become even more frequent, given the great diversity of non-hematophagous bats, as well as the real and constant possibility of transmission between different bat species. These animals have an incredible ability for true flight, which makes them travel great distances, thus facilitating the sharing of territories and favoring the transmission and dissemination of RABV among them.

Ultimately, more genomic surveillance studies, particularly AgV, must be conducted with a larger number and diversity of samples, which will allow a more comprehensive understanding of the correlation between the genetic and ecological characteristics of the RABV variants. This would also enlighten the relationships between the divergent sequences within the established clades, thus strengthening the results of this study. This study highlights the heterogeneity and richness of the genomic information when it comes to viral diseases in the Amazon region, with the remarkable role of rabies in this context.

## Data availability statement

The datasets presented in this study can be found in online repositories. The names of the repository/repositories and accession number(s) can be found below: https://www.ncbi.nlm.nih.gov/genbank/, OP007154; https://www.ncbi.nlm.nih.gov/genbank/, OP007155; https://www.ncbi.nlm.nih.gov/genbank/, OP007156; https://www.ncbi.nlm.nih.gov/genbank/, OP007157; https://www.ncbi.nlm.nih.gov/genbank/, OP007158; https://www.ncbi.nlm.nih.gov/genbank/, OP007159.

## Author contributions

TCu: Methodology, Writing—review and editing. FS: Writing—review and editing, Methodology, Formal analysis. SS: Methodology, Writing—review and editing, Formal analysis. AR: Methodology, Writing—review and editing. FP: Writing—review and editing, Methodology, Formal analysis. LC: Writing—review and editing, Formal analysis. AN: Writing—review and editing, Investigation. IO: Investigation, Writing—review and editing. MB: Investigation, Writing—review and editing. RO: Investigation, Writing—review and editing. DD: Writing—review and editing, Formal analysis, Methodology. TP: Writing—review and editing. TCo: Conceptualization, Project administration, Supervision, Visualization, Writing—original draft.
