# Supplementary material for: Phylogenetic analysis of rabies surveillance samples from north and northeast Brazil
Source: Front Vet Sci. 2023 Sep 29;10:1257558. doi: 10.3389/fvets.2023.1257558 (PMC10570608; doi:10.3389/fvets.2023.1257558)
Supplement: Supplementary file 1 [file Data_Sheet_1.docx]

Supplementary Material

Phylogenetic Analysis of Rabies Surveillance Samples from North and Northeast Brazil

**Taciana Fernandes Souza Barbosa Coelho*, Fábio Silva da Silva, Sandro Patroca da Silva, Ana Cecília Ribeiro Cruz, Francisco Amilton dos Santos Paiva, Livia Medeiros Neves Casseb, Ana de Nazaré Silva do Nascimento, Iza Alencar Sampaio de Oliveira, Marlon de Araújo Castelo Branco, Rodrigo Adolpho Brasil de Oliveira, Darlene de Brito Simith Durans, Thito Yan Bezerra da Paz, Tânia Cristina Alves da Silveira da Cunha.**

*** Correspondence:** Corresponding Author: tacianabarbosa@iec.gov.br

# Supplementary Figures


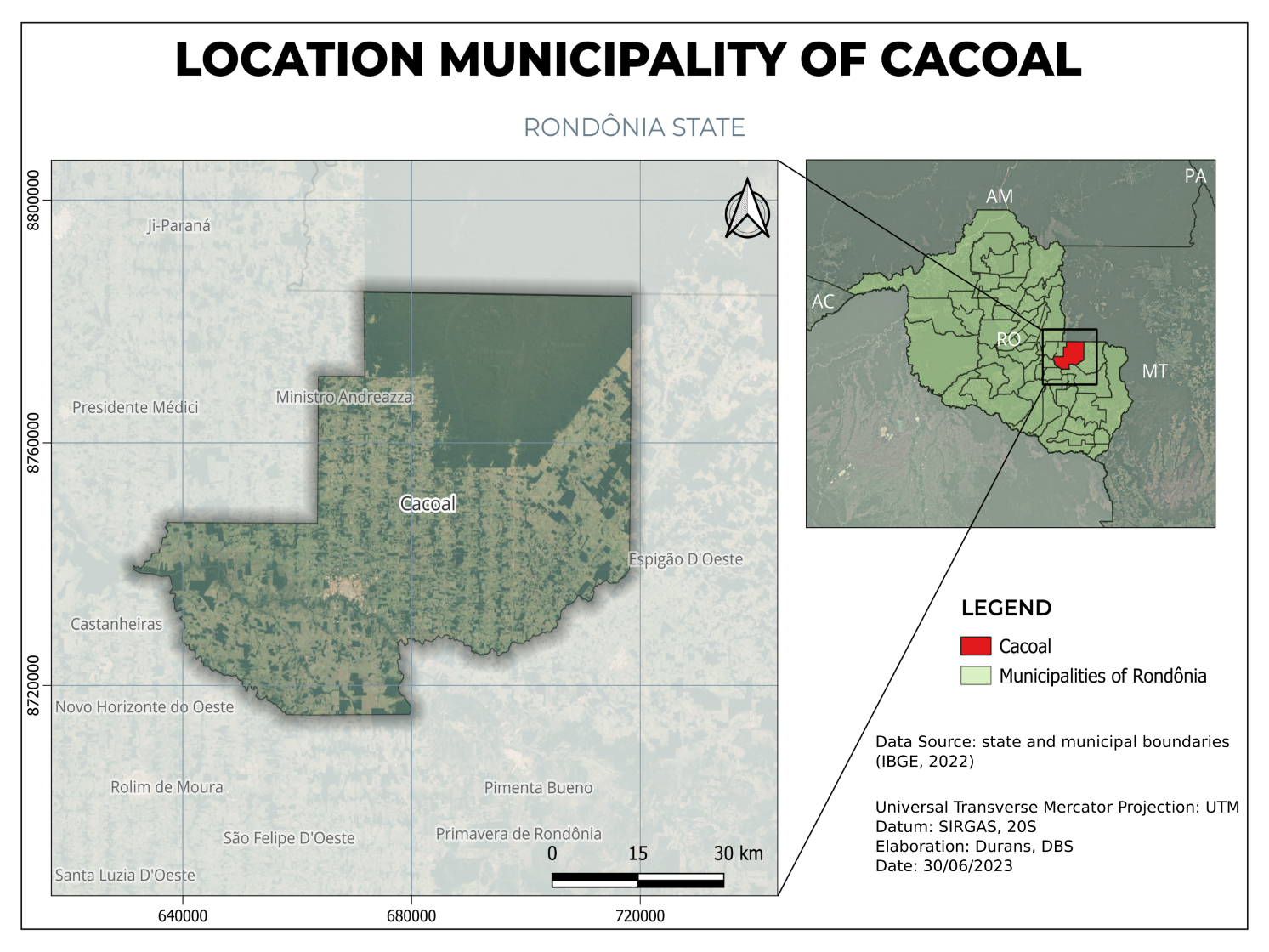


**Supplementary Figure 1.** Location of the municipality of Cacoal-RO where the first description of AgV6 occurred.
